# Psychometric evaluation of a four-item scale derived from the PHQ-9 and GAD-7 among community adults based on multivariate generalizability theory and item response theory

**DOI:** 10.3389/fpsyg.2026.1827821

**Published:** 2026-05-29

**Authors:** Nan Cheng, Chongwei Song, Yang Chen, Jiali Wang, Weiqi Tang, Shifan He, Dejue Men, Jinglin Huang, Wenxiu Zhao, Jianzhong Yin, Qiong Meng

**Affiliations:** 1 School of Public Health, Kunming Medical University, Kunming, China; 2 Yunnan Provincial Key Laboratory of Public Health and Biosafety, Kunming, China; 3 Yunnan Provincial Center for Disease Control and Prevention, Kunming, China; 4 Yunnan Provincial Hospital of Infectious Diseases, Kunming, China, China; 5 School of Public Health, Dali University, Dali, China

**Keywords:** community adults, item response theory, multivariate generalizability theory, PHQ-4, reliability, validity

## Abstract

**Background:**

The Patient Health Questionnaire-4 (PHQ-4) consists of the first two items of the Patient Health Questionnaire-9 (PHQ-9) and the first two items of the Generalized Anxiety Disorder-7 (GAD-7). To date, no studies have examined the psychometric properties of this PHQ-4 in a Chinese community sample using modern measurement theory. This study aims to conduct a preliminary psychometric evaluation of the PHQ-4 among community adults based on modern measurement theory.

**Methods:**

The Chinese version of the combined PHQ-9 and GAD-7 scale (termed the PHQ-16) was used to assess depression and anxiety symptoms among community-dwelling adults residing in rural areas of Yunnan Province, based on the follow up survey of the China Multi-Ethnic Cohort. We selected the items from the PHQ-16 based on the original structure of the PHQ-4 to form a 4-item scale and refer to it as a “PHQ-4-like” scale. The reliability of the PHQ-16 and the PHQ-4-like scale was evaluated using multivariate generalizability theory (MGT). The validity of the PHQ-4-like scale was evaluated using item response theory (IRT).

**Results:**

Based on MGT, reducing the number of items in the PHQ-16 from 16 to 4 decreased the composite generalizability coefficient from 0.906 to 0.719 and the composite reliability index from 0.894 to 0.693. However, when the first two items of the PHQ-9 and the first two items of the GAD-7 were combined to form a four-item scale (the PHQ-4-like scale), the composite generalizability coefficient and composite reliability index were 0.818 and 0.815, respectively. Both the PHQ-9 and the GAD-7 exhibited monotonically increasing threshold parameters. Items 1, 2, 4, and 6 of the PHQ-9 and items 2 and 3 of the GAD-7 showed high discrimination parameters. High average information values were observed for items 1, 2, and 4 of the PHQ-9 and items 2, 3, and 4 of the GAD-7.

**Conclusion:**

The PHQ-4-like scale demonstrated satisfactory reliability and validity in this rural Chinese community sample. However, the PHQ-4 screening performance requires criterion validation. Regarding the anxiety dimension of this scale, it may also be worthwhile to consider combining items 2 and 3 from the GAD-7.

## Introduction

Nearly one-third of people globally have been identified as having experienced at least one common mental disorder in their lifetime (Steel et al., 2014), and anxiety and depression are among the most prevalent disorders. According to WHO estimates for conflict-affected populations, the mean comorbidity-adjusted, age-standardized point prevalence of mild forms of depression, anxiety, and post-traumatic stress disorder was 13.0% (95% CI 10.3–16.2%) (Charlson et al., 2019). A meta-analysis of residents in China found that stress (48.1%), depression (26.9%) and anxiety (21.8%) were the most common mental health consequences of the COVID-19 pandemic (Bareeqa et al., 2021). Anxiety and depression cause serious harm to personal health by interacting with diabetes, cardiovascular disease and other chronic diseases (Buchberger et al., 2016; Seligman and Nemeroff, 2015).

Many instruments have been used to measure depression and anxiety symptoms. The Patient Health Questionnaire-9 (PHQ-9) (Kroenke et al., 2001) is a well-known scale used to screen for depressive symptoms and the Generalized Anxiety Disorder-7 (GAD-7) (Spitzer et al., 2006) is a well-known scale used to screen for anxiety symptoms. Recently, one trend in scale development has been the simplification of existing scales while preserving sufficient information and maintaining good reliability. For example, the WHO Quality of Life-100 scale (WHOQOL-100), a 100-item scale, was simplified into the WHOQOL-BREF, which contains only 26 items (Skevington et al., 2004). Similarly, the Chinese Short Version of the Autism-Spectrum Quotient (AQ-CSV) was developed by simplifying the original 50-item scale to 15 items while preserving its psychometric properties (Xu et al., 2025). Simplification enhances research efficiency by facilitating participants’ concentration and sustained attention during scale completion, thereby reducing the risk for information bias. The PHQ-9 and the GAD-7 also were also simplified by Kurt Kroenke in 2003 and 2007 (Kroenke et al., 2003; Kroenke et al., 2007), respectively, yielding the PHQ-2 and the GAD-2. In 2009, those two ultra-brief scales were combined to form the Patient Health Questionnaire-4 (PHQ-4), and its reliability and validity were subsequently evaluated in a sample of 2149 patients across 15 primary care clinics (Kroenke et al., 2009). The PHQ-4 is recognized as an ultra-brief, reliable, and valid instrument for screening depression and anxiety symptoms—primarily used in clinical settings but also increasingly applied in community populations. For instance, studies conducted among community residents in the Philippines (Mendoza et al., 2022) and Colombian households (Kocalevent et al., 2014) reported Cronbach’s alpha of 0.82 and 0.84, respectively. The PHQ-4 has also been demonstrated to be reliable and valid in the general population of Greece (Christodoulaki et al., 2022). In 2021, a research team validated the Chinese version of the PHQ-4, confirming its reliability and validity for screening depression and anxiety symptoms among outpatients at Chinese community healthcare centers (Qian et al., 2021).

However, existing research on the reliability and validity evaluation of the PHQ-4 has relied exclusively on classical test theory (CTT). CTT estimates reliability based on a single error source, thereby ignoring other potential sources of measurement error variance. When multiple sources contribute to measurement errors, CTT may not be sufficient. Moreover, CTT suffers from several well-documented limitations: item parameters are sample-dependent and the metric scale for person ability (i.e., latent trait level) and item difficulty are not consistent. Modern measurement theory was developed to address these shortcomings. It includes generalizability theory (GT) and Item Response Theory (IRT). GT provides a comprehensive and unifying framework that extends beyond the CTT model—which posits a single error term—by enabling simultaneous analysis of main effects and interaction effects as sources of error variance. Applications of GT include the univariate generalizability theory (UGT) approach and the multivariate generalizability theory (MGT) approach. MGT was initially proposed by Cronbach based on multivariate analysis of variance (MANOVA), and is appropriate for multidimensional and complex measurement situations. In MGT, the reliability of all domains is estimated simultaneously, rather than for each domain in isolation. IRT was initially known as Latent Trait Theory in its early developmental stages. It adopts a microscopic perspective, selecting items for retention or deletion based on item-level indicators—such as discrimination, difficulty, and information. As mentioned above, the evaluation of the reliability and validity of the Chinese version of the PHQ-4 has primarily been conducted among outpatients at community healthcare centers. To date, no studies have examined the psychometric properties of the Chinese version of the PHQ-4 based on modern measurement theory.

The main aim of this study was to evaluate the reliability of a 4-item scale—termed the PHQ-4-like scale—which was derived by selecting items from the PHQ-9 and the GAD-7 according to the original structure of the PHQ-4, among community-dwelling adults based on MGT and to assess its validity based on IRT. These evaluations were conducted to provide empirical support for using the PHQ-4 to assess depression and anxiety symptoms among community-dwelling adults.

## Materials and methods

### Participants

This study surveyed a total of 2333 community residents aged between 30 and 79 years old who lived in Yunnan Province, based on the first follow-up survey of the China Multi-Ethnic Cohort (CMEC). The CMEC was established in five provinces (Chongqing, Guizhou, Sichuan, Tibet and Yunnan) in Southwest China. The baseline survey was completed through multistage stratified cluster sampling between May 2018 and September 2019 (Zhao et al., 2021). Eligible participants were aged 30 years or older and were able to read and understand the questionnaires. Individuals diagnosed with severe physical or mental illnesses (e.g., schizophrenia or bipolar disorder) were excluded. The first follow-up survey of the CMEC was conducted in 2020 by randomly selecting 10% of participants who had completed the baseline survey. Power analysis indicated statistical power of 1.00 (*F* = 0.25, α = 0.05) for the present study (total sample size = 2333).

### Measurement scale

For the sake of discussion, the combined scale of the PHQ-9 and the GAD-7 was named as the PHQ-16. Thus, the PHQ-16 consists of two domains: depression and anxiety. The depression domain consists of 9 items, which are used to measure interest, sentiment, sleep, energy, appetite, remorse, attention, slowness, and suicide separately. The anxiety domain consists of 7 items, which are used to measure tension, concern, worry, relaxation, restlessness, testiness and fear. Each item was answered with a 0-3 Likert and was given a score ranging from 0 to 3. The sum of the scores of each item is the total score of the scale, which ranges from 0 to 48. More details are presented in Supplementary Table 1.

As mentioned previously, the PHQ-4 comprises the PHQ-2 and the GAD-2. The two items constituting the PHQ-2 are the first two items of the PHQ-9, whereas the two items constituting the GAD-2 are the first two items of the GAD-7. The PHQ-4 consists of two domains: the depression domain and the anxiety domain. Four items separately measure the following symptoms: (1) little interest or pleasure in doing things, (2) feeling down, depressed, or hopeless, (3) feeling nervous, anxious, or on edge, and (4) being unable to stop or control worrying. Each item is rated on a 0-3 Likert scale (“Not at all,” “Several days,” “More than half the days,” and “Nearly every day”). The total score of the PHQ-4 ranges from 0 to 12.

In MGT, a single set of measurement data can be used to simultaneously estimate reliability coefficients for different test lengths (i.e., different numbers of items). For example, by administering only the 16-item scale, we can calculate the reliability coefficients of both the full 16-item scale and its 4-item subset using MGT. Only by using the data obtained from the PHQ-16 as the analytical input can we calculate and compare the reliability coefficients of the 4-item version (derived by reducing the item count from 16 to 4) and those of the PHQ-4 in order to verify that the PHQ-4 is a reliable and appropriate simplified version. Therefore, the measurement scale used in this study was the PHQ-16, rather than the PHQ-4.

We selected the items from the PHQ-16 based on the original structure of the PHQ-4 to form a 4-item scale and refer to it as a “PHQ-4-like” scale. The PHQ-4–like scale scores were extracted from the PHQ-16 data.

### Measurement process

The investigators were the trained medical students from local medical universities who were familiar with the local minority languages. On the day of data collection, the purpose and objectives of the study were clearly explained to the respondents. A self-developed application (named “CMES App”) with an integrated audio recording function was used to conduct the survey via face-to-face interviews. A self-developed structured questionnaire was used to collect the demographic information about the participants, including gender, age group (30–39, 40–49, 50–59, 60–69, and 70–79 years), ethnic group (Han, Yi, Bai), education level (primary school or below, junior high school, high school or above), marital status (married or cohabiting, separated or divorced, widowed, unmarried), and occupation (agricultural or fishery worker, other). The Chinese version of PHQ-16 was used to measure the depression and anxiety symptoms among community residents. All procedures were conducted in strict accordance with relevant guidelines and regulations. Written informed consent was obtained from all participants prior to their involvement in the study. This research received ethical approval from the Kunming Medical University Medical Ethical Review Board (KMMU2020MEC078).

### Evaluation of reliability based on MGT

GT contains two stages: Generalizability Study (G-study) and Decision Study (D-study). The G-study serves as a “pilot” study that decomposes the variance and covariance components related to various error sources to help confirm the relationship between the measurement goal and measurement facets using analysis of variance (ANOVA) or multivariate analysis of variance (MANOVA). In the D-study, the information from the G-study is used for the planning of an “optimal” measurement protocol so that the best possible reliability can be achieved while balancing other factors.

In the GT, the measurement target refers to the object or phenomena that is being measured, such as a person’s intelligence, their attitude toward a particular topic, or their ability to perform a specific task. The measurement facet refers to the different aspects or sources of variability that can affect the measurement process, such as the different raters who are scoring a test, the different items that are being used to assess a trait, or the different occasions on which a test is administered. In the current study, the measurement target is the depression and anxiety symptoms of “persons,” which is abbreviated as “*p*,” and the “items” facet (abbreviated as “*i*”) is the only facet of measurement because the depression and anxiety symptoms are self-report measurements without rater facet. The *i* facet is a random facet because a different set of items can be involved in each response. The data are unbalanced because there are unequal numbers of items within each domain. The domains facet (abbreviated as “*h*”) is treated as the fixed facet because every replication of the measurement of the depression and anxiety symptoms involved the same domains. It is defined that facet *i* was nested within facet *h*. In addition, facet *p* completely overlaps with facet *i* because every person needs to answer every item in the PHQ-16. We can write this design as p=(i:h) where *p*, *i*, and *h* represent persons, items, and domains facets, respectively.

In the multivariate G-study, the variance-covariance component matrix among persons (Σ_p_), the variance-covariance component matrix for items within a domain (Σ_i_) and the patient-item interaction within a domain (Σ_pi_) were calculated. Once the variance and covariance matrix from the G-study results are available, they can be used in the D-study to estimate the variance components of the universe score and the variance components of the measurement error, including relative error variance (σ^2^(δ)) and absolute error variance (σ^2^(Δ)), and then further to calculate the two reliability coefficients, including the generalizability coefficient (*G*) and reliability index (Φ). σ^2^(δ) reflects the total variation in all relevant interaction effects with the measurement goal, and σ^2^(Δ) represents the total variation in all effects except the measurement goal itself (Zhi Ming and Lei, 2003). *G* reflects the proportion of effective variation to the sum of effective variation and σ^2^(δ), and Φ reflects the proportion of effective variation to the sum of effective variation and σ^2^(Δ). The effective variation is usually reflected by the variance components of the measurement goal, which is also called the universe score variance [σ^2^(*P*)]. The relationships between the measurement error variances and the reliability coefficients are as follows (Equations 1, 2):


$$G=\sigma^{2}\,\left(P\right)/\left[\sigma^{2}\,\left(P\right)+\sigma^{2}\,\left(\delta \right)\right]$$ (1)

$$\Phi =\sigma^{2}\,\left(P\right)/\left[\sigma^{2}\,\left(P\right)+\sigma^{2}\,\left(\Delta \right)\right]$$ (2)

In the MGT framework, both the above mentioned indexes for every domain of scale and the corresponding composite values of these indexes for the overall scale can be obtained by defining a weight coefficient (*w_h_*). The weight coefficient (*w_h_*) reflects the proportion of the number of items in each domain to the total number of items in the overall scale.

A series of analyses based on MGT for the PHQ-16 were performed. The variance-covariance components and the proportion of the variance-covariance of each facet were calculated in the G-study of MGT. The indexes that reflect the measurement reliability were calculated in the D-study of MGT, including σ^2^(*P*), σ^2^(δ), σ^2^(Δ), error variance for mean ($\sigma_{X\,P\,I}^{2}$), *G*, Φ, composite *G*, and composite Φ.

Several D-studies under the original measurement protocol and the new measurement protocols modified by changing the number of items were analyzed to understand how the measurement reliability could vary with a changing number of items. MGT analyses were performed using mGENOVA 2.1 (Brennan, 2001).

We can use the D-Studies to evaluate the reliability of the measurement model and then adjust the design of the D-Studies based on the merits of their results to further examine the impact of changes in different facets on reliability. Therefore, to assess whether the reliability of the scale remained acceptable after reducing the number of items in the depression domain from 9 to 2 and in the anxiety domain from 7 to 2, a multivariate D study was conducted.

To determine whether the four items in the PHQ-4-like scale were more appropriate than other items in the PHQ-16, we used GT to evaluate the reliability of every possible two-item combination within each domain of the PHQ-16 (a combined scale comprising the PHQ-9 and the GAD-7). Specifically, all pairwise combinations of two items from the PHQ-9 were formed to construct a two-item depression domain, yielding 36 possible combinations. D-studies were conducted for each. Similarly, any two items were selected from the 7 items of the GAD-7 and combined into a two-item anxiety domain. Then, D-Studies were conducted for all 21 possible combinations.

### Evaluation of validity based on IRT

IRT uses a mathematical function, the item characteristic curve (ICC), to describe the relationship between a subject’s latent trait level and their probability of responding to an item in a particular way (Hays et al., 2000). The threshold parameter (*b)* is the abscissa value at the inflection point of the ICC, where the probability of a correct response is 0.50. The discrimination parameter (*a*) is the slope of the ICC at that inflection point; higher values of a indicate better differentiation among examinees with different ability levels. Items with higher discrimination are more effective in differentiating ability levels. According to relevant research recommendations, the discrimination parameter should range from 0 to 2.8, and the difficulty parameter should range from −3 to +3 (Baker, 2001). If an item characteristic curve is too flat, the item cannot effectively differentiate among different levels of the latent trait. If the item characteristic curves overlap significantly, it suggests that the threshold parameters have little difference. When evaluating a subject’s latent trait level, the amount of information contributed by each item is defined as the item information function. The sum of item information functions yields the test information function. The larger the area under the test information curve, the more information is provided across a wider ability range.

Because the PHQ16 utilized Likert-scale scoring and self-reported measures by patients who were generally not engaged in guessing, we selected the graded response model. Each item in the graded response model had one discrimination parameter and (*k*-1) threshold parameters, where *k* is the number of answer options for the item. In this study, the response options for each item of the scale were of four grades. Therefore, each item had three threshold parameters. For clarity, we refer to them respectively as response threshold 1 (*b*1), response threshold 2 (*b*2), and response threshold 3 (*b*3). IRT analyses were performed using MULTILOG 7.03.

## Results

### Basic demographic characteristics of the participants

Data were collected from 2,333 residents aged 30–79 from CMEC, among whom 702 were male (30.09%) and 1631 were female (69.91%). The majority of participants were middle-aged, aged 40–60 years, with the highest proportion in the 50–60 age group (42.33%). Ethnic groups included Han, Yi, and Bai, with Han accounting for the highest proportion (42.43%). Regarding marital status, the majority were married (90.87%). With respect to the education level, the largest proportion had completed primary school or below (69.57%), 549 had completed junior high school (23.53%), and 161 had completed high school or above (6.90%). Regarding occupation, the largest proportion was agricultural or fishery workers (69.07%). More details are presented in Supplementary Figure 1.

### Reliability

The results of the G-study for the PHQ-16 (Table 1) in MGT showed that the estimated variance-covariance components associated with facet *i* were smallest in two domains. The results of the D-study for the PHQ-16 showed that *G* and Φ in the depression domain were greater than 0.7 but less than 0.8, and *G* and Φ in the anxiety domain were greater than 0.8 but less than 0.9. After reducing the number of items per domain, the two reliability coefficients (*G* and Φ) decreased for both the depression and anxiety domains. The anxiety domain was less affected by simplification than the depression domain because the two reliability coefficients of the depression domain were greater than 0.4 but less than 0.5, but the two reliability coefficients of the anxiety domain were greater than 0.6 but less than 0.8. When the number of items of the PHQ-16 was reduced from 16 to 4, the composite generalizability coefficient (*G*) decreased from 0.906 to 0.719, and the composite reliability index (Φ) decreased from 0.894 to 0.694 (Table 2).

**TABLE 1:** The estimated variance-covariance components for *P* = (i:h) sign in the G-study for the PHQ-16.

Measurement facets	Domains of the PHQ-16	
	Depression	Anxiety
Person (*p*)	0.0998^a^	0.8169^b^
	0.1041^c^	0.1627^a^
Item (*i*)	0.0422a	–
	–	0.0061^a^
Person × Item (*pi*)	0.2297^a^	–
	–	0.1384^a^

*^a^*Diagonal elements are estimated variance components and are presented in bold type.

*^b^*The upper diagonal elements are correlations.

*^c^*The lower diagonal elements are covariances.

**TABLE 2:** D-Studies with the original test length and adjusted D-Study for the PHQ-16.

Index	PHQ-16 with the original test length		Adjusted D-studies	
	Depression	Anxiety	Depression (${n^{\prime }}_{i}=2$)	Anxiety (${n^{\prime }}_{i}=2$)
$\sigma_{p}^{2}$	0.0998	0.1627	0.0998	0.1627
$\sigma_{\delta }^{2}$	0.0255	0.0198	0.1148	0.0692
$\sigma_{\Delta }^{2}$	0.0302	0.0206	0.1359	0.0722
$\sigma_{X\,P\,I}^{2}$	0.0047	0.0009	0.0212	0.0031
*G*	0.7964	0.8917	0.4651	0.7016
Φ	0.7678	0.8875	0.4235	0.6926
Composite *G*	0.9058		0.7190	
Composite Φ	0.8941		0.6935	

$\sigma_{p}^{2}$, Universe score variance; $\sigma_{\delta }^{2}$, Relative error variance; $\sigma_{\Delta }^{2}$, Absolute error variance; $\sigma_{X\,P\,I}^{2}$, Error variance for mean; *G*, Generalizability Coefficient; Φ, Reliability index.

The results of the G-study showed that the estimated variance-covariance components from *i* were smallest in two domains (Table 3). The D-study showed that both the *G* and Φ of the two domains were greater than 0.70 but less than 0.80. Both the composite *G* and composite Φ of the total scale were greater than 0.80 (Table 4). The two composite reliability coefficients of the PHQ-4-like scale were higher than those calculated when the number of items of the PHQ-16 was adjusted from 16 to 4.

**TABLE 3:** The estimated variance-covariance components for *P* = (*i*:*h*) design in the G-study for the PHQ-4.

Measurement facets	Domains of the PHD-4	
	Depression	Anxiety
Person (*p*)	**0.1869** ^a^	0.7044^b^
	0.1321^c^	**0.1882** ^a^
Item (*i*)	**0.0059** ^a^	**-**
	**-**	**0.0003** ^a^
Person × Item (*pi*)	**0.1454** ^a^	**-**
	**-**	**0.1386** ^a^

^a^Diagonal elements are estimated variance components and are presented in bold type.

^b^The upper diagonal elements are correlations.

^c^The lower diagonal elements are covariances.

**TABLE 4:** D-Studies with the original test length for the PHQ-4.

Domain	$\sigma_{p}^{2}$	$\sigma_{\delta }^{2}$	$\sigma_{\Delta }^{2}$	$\sigma_{X\,P\,I}^{2}$	*G*	Φ
Depression	0.1869	0.0727	0.0757	0.0030	0.7199	0.7118
Anxiety	0.1882	0.0693	0.0694	0.0003	0.7309	0.7305
Total Scale	0.1598	0.0355	0.0363	0.0009	0.8182	0.8150

$\sigma_{p}^{2}$, Universe score variance; $\sigma_{\delta }^{2}$, Relative error variance;$\sigma_{\Delta }^{2}$, Absolute error variance; $\sigma_{X\,P\,I}^{2}$, Error variance for mean; *G*, Generalizability Coefficient; Φ, Reliability index.

There were 4 combinations for which both *G* and Φ were higher than 0.6 (item 1 + item 2, item 1 + item 4, item 2 + item 4, and item 7 + item 8) and only 1 combination for which both *G* and Φ were higher than 0.7 (item 1 + item 2) among the 36 combinations of the PHQ-9 (Supplementary Table 2). The structure of the scale under this combination matched the exact structure of the PHQ-2. Both *G* (0.7199) and Φ (0.7118), which constituted the PHQ-2, were greatest among all combinations. There were 19 combinations for which both *G* and Φ were higher than 0.6 and 10 combinations for which both *G* and Φ were higher than 0.7 among 21 combinations of the GAD-7 (Supplementary Table 3). If G was selected as the index of responding reliability and the generalizability coefficients of the 21 combinations were ranked from the largest to the smallest, *G* of the combination constituting the GAD-2 was ranked sixth. If Φ was selected as the index of responding reliability and those of the 21 combinations were ranked from the largest to the smallest, Φ of the combination constituting the GAD-2 was ranked fifth.

### Validity

According to Tables 5, 6, the discrimination parameters (*a*) of all items in the PHQ-9 ranged from 0.0 to 2.8. The discrimination parameters of items 2 and 3 in the GAD-7 exceeded 2.8. The threshold parameters (*b*) of all items in the GAD-7 and the PHQ-9 showed an increasing trend. For items 8 and 9 in the PHQ-9 and items 5 and 7 in the GAD-7, the threshold parameter 3 (*b3*) was relatively high. The average information of the PHQ-9 ranged from 0.27 to 0.93, with the top three values found in item 1, item 2, and item 4. In the GAD-7, the average information values of the items ranged from 0.67 to 1.72, with the top three values observed in item 2, item 3, and item 4.

**TABLE 5:** Item analyses of the PHQ-9 based on item response theory.

Index	Item1	Item2	Item3	Item4	Item5	Item6	Item7	Item8	Item9
*a*	2.43	2.63	1.11	2.34	1.74	2.37	1.77	1.70	1.89
*b1*	0.57	0.24	−0.02	−0.05	0.94	1.06	0.89	0.95	1.66
*b2*	2.28	2.09	1.57	1.84	3.00	2.69	3.06	3.12	3.31
*b3*	2.96	2.82	2.84	2.54	3.99	3.36	3.82	4.15	4.29
Average information	0.78	0.93	0.27	0.83	0.39	0.64	0.40	0.37	0.36
Maximum information	1.74	2.00	0.38	1.61	0.85	1.66	0.88	0.79	0.95

*a*, discrimination parameters;

*b*, threshold parameters of different response category for each item.

**TABLE 6:** Item analyses of the GAD-7 based on item response theory.

Index	Item1	Item2	Item3	Item4	Item5	Item6	Item7
*a*	3.00	3.70	3.98	3.77	2.71	2.76	2.30
*b1*	0.20	0.30	0.23	0.46	0.57	0.12	0.76
*b2*	2.02	1.77	1.70	2.11	2.33	1.93	2.45
*b3*	2.92	2.56	2.50	2.85	3.21	2.90	3.32
Average information	1.12	1.56	1.72	1.49	0.88	1.02	0.67
Maximum information	2.41	3.62	4.11	3.79	2.03	2.04	1.52

*a*, discrimination parameters;

*b*, threshold parameters of different response category for each item.

**FIGURE 1:**
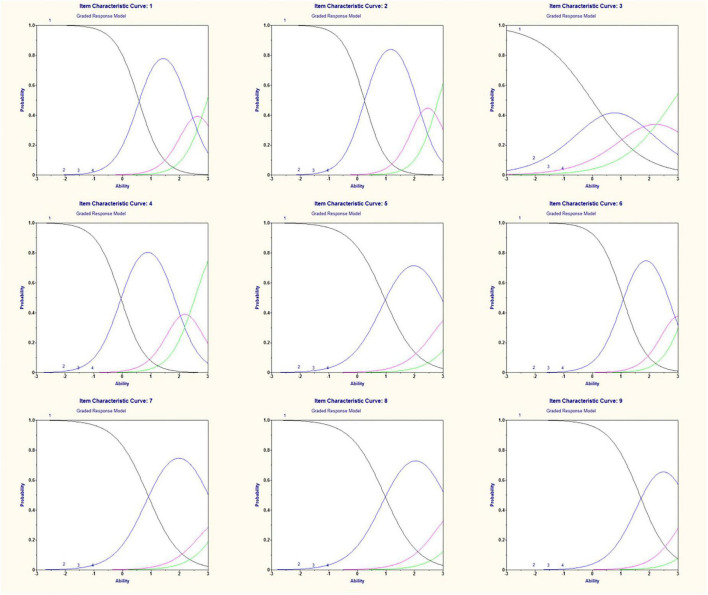
The item characteristic curve of the 7 items for the GAD-7 scale.

**FIGURE 2:**
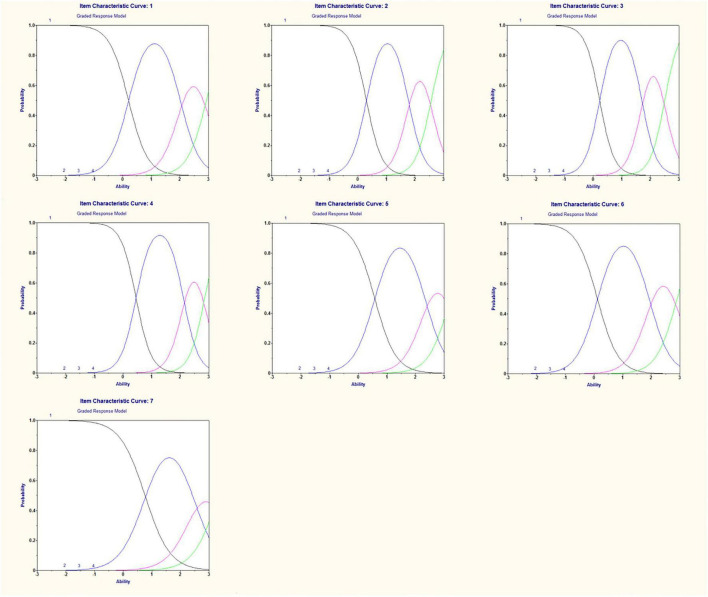
The item characteristic curve of the 9 items for the PHQ-9.

The scale used a 4-point rating system. Each item had four curves reflecting the probability of selecting each response option. Each curve represented a response option and indicated the probability of selecting that option at specific levels of depression or anxiety. Item characteristic curves that were distributed on the right side of the figure indicated higher threshold levels. In the PHQ-9, the item characteristic curves of items 1, 2, and 4 were closer to the standard curves. Their item information curves covered a broader range (Figure 1). In the GAD-7, item characteristic curves for items 1, 2, and 3 had larger coverage areas (Figure 2). The test information curves suggested that the expected scores would increase with higher levels of depression or anxiety. The results for both the item and test information curves were consistent with the parameter estimates (see Supplementary material for more information).

## Discussion

Our study used two modern measurement theories to assess whether the PHQ-4-like scale had satisfactory reliability and validity. The conventional reliability approaches in CTT are typically post hoc, meaning that measurement reliability is calculated after the fact. In contrast, GT can be employed proactively to design more effective measurement protocols. In GT, *G* and Φ coefficients are two important reliability coefficients, which are used to depict the reliability for “relative decision” and “absolute decision,” respectively. Within the GT framework, “relative decision” depends on norm-referenced score interpretation which is concerned with the consistency of relative standings of the individuals rather than the consistency of the actual scores, whereas “absolute decision” depends on criterion-referenced score interpretation which is concerned with both the consistency of the relative standings of the individuals and the consistency of the actual scores (Schünemann et al., 2007). The coefficients are selected depending on the researchers’ interests. If one’s interest lies in conducting a norm-referenced measurement to compare the level of depression and anxiety symptoms of different persons (relative decision), a *G* coefficient should be selected to quantify the dependability of the score. If one’s goal is to perform a criterion-referenced test to investigate the level of depression and anxiety symptoms of measurement objects (absolute decision), a Φ coefficient should be used to inform about how dependable a score is. It is clear that the Φ coefficient is typically lower than *G* coefficient for every domain because the variance component of item within domain (*i:h*) is factored into the absolute error variance and Φ coefficient but not the relative error variance and *G* coefficient. When we use the scale to screen anxiety and depression symptoms, it should be an absolute decision, so it is more appropriate to choose the Φ coefficient.

High reliability is a necessary condition for high validity. Regarding reliability standards, researchers have slightly different choices for determining the standard to use when evaluating reliability coefficients. In a medical education assessment manual, reliability coefficients between 0.50 and 0.70 may be sufficient for multiple assessments. A higher reliability standard of greater than 0.80 should be sought when it is a high-stakes assessment. For very high-stakes tests, such as professional licensing, in which the term “stakes” refers to “the importance of the results of testing programs for individuals, institutions, or groups,” higher than 0.9 is generally recommended (Peeters and Cor, 2020). In the studies on the Chinese version of the Meaning in Life Questionnaire’s (MLQ) scales (Chen and Gao, 2021) and the Chinese version of the Functional Assessment of Cancer Therapy—Leukemia’s (FACT-Leu) scales (Zhou et al., 2026), reliability coefficients higher than 0.80 were considered the reference standard for good reliability. Therefore, we have adopted a standard of higher than 0.80 as the “great” index, while an acceptable range would be between 0.70 and 0.80. Finally, any value below 0.70 is considered to indicate poor reliability and is not recommended for practical use.

When the number of items of each domain was adjusted to 2, both *G* and Φ of the depression domain were larger than 0.4 but smaller than 0.70, *G* and Φ of the anxiety domain were close to 0.70, composite *G* was greater than 0.70 and composite Φ was close to 0.70. Moreover, the two reliability coefficients were smaller than those of the PHQ-4-like scale. This means that the reliability of the two-item simplified scale cannot be guaranteed by selecting any two items during the simplification process of the PHQ-16. In the study, both the composite *G* and composite Φ of the PHQ-4-like scale were larger than 0.80. This means that the reliability of the PHQ-4-like scale remains “great.” Even at the domain level, the *G* and Φ coefficients of the two domains (depression domain and anxiety domain) of the PHQ-4-like scale were acceptable. Therefore, we can verify that the PHQ-4-like scale is a reliable and appropriate simplified version for the PHQ-16. A recent systematic review of 26 studies reported that the PHQ-4-like scale has adequate internal consistency, with Cronbach’s α ranging from 0.72 to 0.88 (Chen and Gao, 2021). Our results show that the composite G coefficient of the PHQ-4-like scale was 0.818, suggesting that the PHQ-4-like scale is also reliable in our rural Chinese sample from Yunnan.

According to the IRT analysis, items 1 and 2 of the PHQ-9 demonstrated superior performance compared to other items in terms of discrimination and average information, indicating their effectiveness in distinguishing individuals with varying levels of depression and providing more information in measuring depressive symptoms. Similarly, items 1 and 2 of the GAD-7 also exhibited better performance than other items in terms of discrimination and average information, indicating their effectiveness in distinguishing individuals with varying levels of anxiety and providing more information in measuring anxiety symptoms. These findings contribute to the evidence supporting the reliability and validity the PHQ-4-like scale. However, cultural differences may affect symptom expression, and item performance may vary across cultural contexts (Meng et al., 2017; Tang et al., 2024). Therefore, more external validation is needed.

On the other hand, based on the results of reliability and validity, there is an alternative possibility in the anxiety dimension of the PHQ-4. In terms of validity in the GAD-7 item 3 provided higher information than item 1, suggesting that the combination of items 2 and 3 of the GAD-7 could more comprehensively reflect the severity of anxiety symptoms, a finding consistent with previous studies on the GAD-7 (Zhang et al., 2021). In terms of reliability, the combination of items 2 and 3 in the GAD-7 performed the best among the 21 combinations. As mentioned by the developers of GAD-7 (Spitzer et al., 2006), the 2 core criteria A and B (total including A, B, and C) of the DSM-IV (Diagnostic and Statistical Manual of Mental Disorders-4th Edition) definition of the GAD-7 are captured by the first 3 items of the scale. Criterion B was mainly measured by item 2, and criterion A was mainly measured by items 1 and 3. Item 3 assesses “worrying too much about different things” and shows psychological subjective sensation compared with item 1, which assesses “feeling nervous, anxious or on edge.” Therefore, we thought items 2 and 3 could also be good choices to guarantee criteria A and B in terms of the two-item anxiety scale. Although the reliability of the traditional GAD-2 was not the highest in our sample, it remained at an acceptable level, and its content coverage may be broader than that of items 2 and 3. Therefore, we maintain that the value of the traditional GAD-2 is not undermined. At the same time, we acknowledge that anxiety may manifest differently across different contexts and populations (Song et al., 2023), and our findings may not be directly generalizable to other groups.

The innovation of this study was that it was the first to use MGT to evaluate the Chinese version of the PHQ-4-like scale. Several limitations should be noted. This study does not constitute a formal validation of the original PHQ-4, as the PHQ-4 items were derived from the PHQ-9 and GAD-7 responses rather than being administered independently. Independent validation using direct administration of the PHQ-4 is needed. Furthermore, we considered only the item facet and did not explore other measurement facets, such as ethnicity. Because our sample was confined to Yunnan Province, the external validity of the scale remains undetermined. This study used a 10% follow-up subsample; although randomly selected, follow-up selection bias may still be present. Our sample consisted predominantly of females with low educational levels, and included only Han, Yi, and Bai ethnic groups. Therefore, the generalizability of our findings is limited to rural community-dwelling adults aged 30–79 years in Yunnan Province and should not be overgeneralized.

## Conclusion

The PHQ-4-like scale demonstrated satisfactory reliability and validity in this rural Chinese community sample. However, the PHQ-4 screening performance requires criterion validation. Regarding the anxiety dimension of this scale, it may also be worthwhile to consider combining items 2 and 3 from the GAD-7.

## Data Availability

The original contributions presented in this study are included in the article/Supplementary material, further inquiries can be directed to the corresponding authors.
