# Supplementary material for: Psychometric evaluation of a four-item scale derived from the PHQ-9 and GAD-7 among community adults based on multivariate generalizability theory and item response theory
Source: Front Psychol. 2026 May 29;17:1827821. doi: 10.3389/fpsyg.2026.1827821 (PMC13260393; doi:10.3389/fpsyg.2026.1827821)
Supplement: Supplementary file 1 [file Table_1.docx]

Supplementary Material

Table S1 The meaning of each item of PHQ-16 and the distribution of the item scores

| Domains | Number | Subject | Item | Score | | | |
| --- | --- | --- | --- | --- | --- | --- | --- |
|  |  |  |  | 0 | 1 | 2 | 3 |
| Depression | 1 | interest | Little interest or pleasure in doing things | 1603 | 666 | 47 | 17 |
|  | 2 | sentiment | Feeling down, depressed, or hopeless | 1371 | 879 | 63 | 20 |
|  | 3 | sleep | Trouble falling or staying asleep, or sleeping too much | 1158 | 725 | 305 | 145 |
|  | 4 | energy | Feeling tired or having little energy | 1141 | 1043 | 105 | 44 |
|  | 5 | appetite | Poor appetite or overeating | 1772 | 522 | 31 | 8 |
|  | 6 | remorse | Feeling bad about yourself — or that you are a failure or have let yourself or your family down | 1904 | 398 | 23 | 8 |
|  | 7 | attention | Trouble concentrating on things, such as reading the newspaper or watching television | 1747 | 552 | 24 | 10 |
|  | 8 | slowness | Moving or speaking so slowly that other people could have noticed? Or the opposite — being so fidgety or restless that you have been moving around a lot more than usual | 1769 | 529 | 28 | 7 |
|  | 9 | suicide | Thoughts that you would be better off dead or of hurting yourself in some way | 2093 | 222 | 15 | 3 |
|  |  |  |  |  |  |  |  |
| Anxiety | 1 | tension | Feeling nervous, anxious or on edge | 1412 | 858 | 53 | 10 |
|  | 2 | concern | Not being able to stop or control worrying | 1499 | 752 | 66 | 16 |
|  | 3 | worry | Worrying too much about different things | 1440 | 804 | 72 | 17 |
|  | 4 | relaxation | Trouble relaxing | 1626 | 669 | 30 | 8 |
|  | 5 | restlessness | Being so restless that it is hard to sit still | 1679 | 616 | 31 | 7 |
|  | 6 | testiness | Becoming easily annoyed or irritable | 1339 | 910 | 71 | 13 |
|  | 7 | fear | Feeling afraid as if something awful might happen | 1780 | 512 | 33 | 8 |


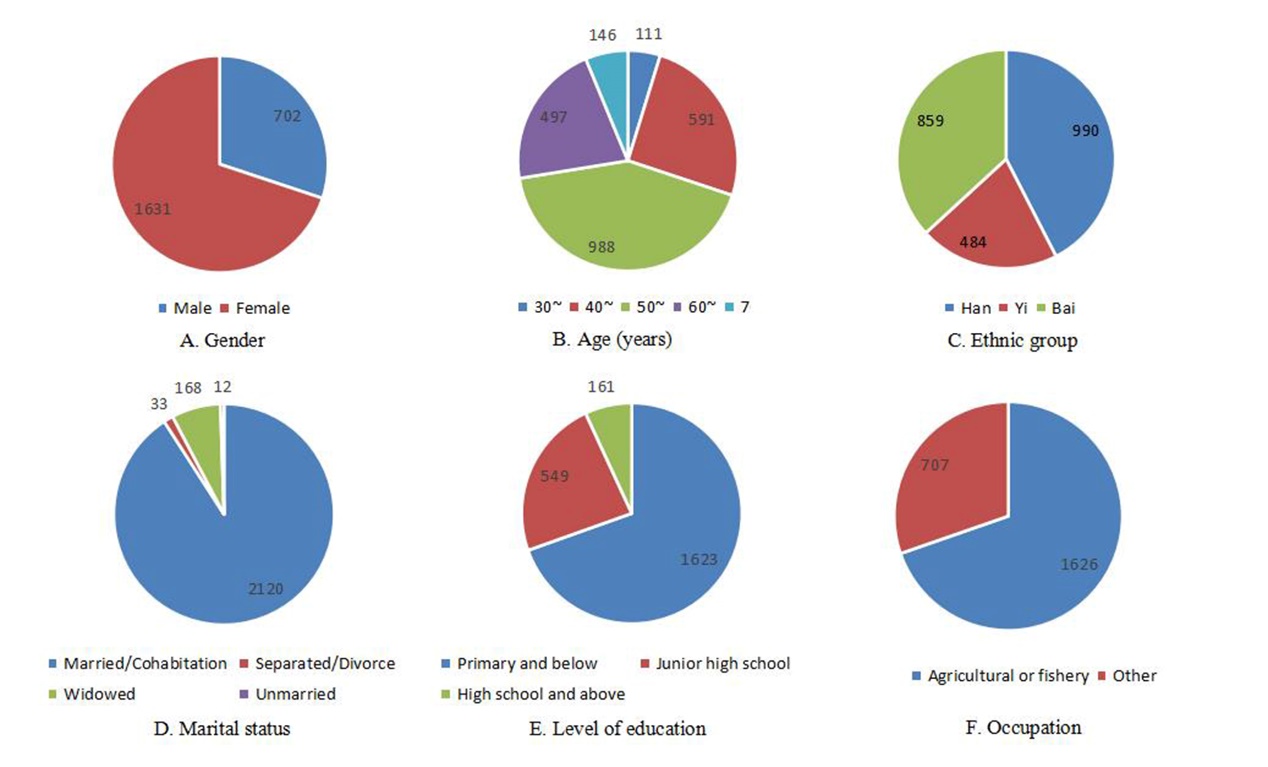


Figure.S1 The distribution of general characteristics of study subjects

Table S2. GT D-Studies of two items combined from PHQ-9

| Number | Item 1 | Item 2 | Item 3 | Item 4 | Item 5 | Item 6 | Item 7 | Item 8 | Item 9 |
| --- | --- | --- | --- | --- | --- | --- | --- | --- | --- |
| Item 1 | - | **0.7199** | 0.4045 | 0.6525 | 0.4724 | 0.5340 | 0.4707 | 0.4864 | 0.3327 |
| Item 2 | **0.7118** | - | 0.4591 | 0.6746 | 0.5076 | 0.5535 | 0.4742 | 0.4780 | 0.4156 |
| Item 3 | 0.3615 | 0.4332 | - | 0.5282 | 0.3311 | 0.2832 | 0.2902 | 0.3183 | 0.1933 |
| Item 4 | 0.6191 | 0.6643 | 0.5200 | - | 0.4915 | 0.4809 | 0.4966 | 0.5104 | 0.3405 |
| Item 5 | 0.4676 | 0.4844 | 0.2770 | 0.4381 | - | 0.5400 | 0.4837 | 0.4402 | 0.4360 |
| Item 6 | 0.5181 | 0.5100 | 0.2244 | 0.4069 | 0.5369 | - | 0.5563 | 0.5290 | 0.5381 |
| Item 7 | 0.4670 | 0.4541 | 0.2434 | 0.4457 | 0.4837 | 0.5521 | - | 0.6251 | 0.4284 |
| Item 8 | 0.4814 | 0.4553 | 0.2656 | 0.4548 | 0.4402 | 0.5260 | 0.6251 | - | 0.4284 |
| Item 9 | 0.3005 | 0.3468 | 0.1378 | 0.2539 | 0.4161 | 0.5287 | 0.4065 | 0.4065 | - |

1. Upper diagonal elements are *G*. Lower diagonal elements are *Ф*.

2. The values higher than 0.70 are presented in bold type

3. The two reliability coefficients of the combination constituting PHQ-2 are underlined.

Table S3. GT D-Studies of two items combined from GAD-7

| Number | Item 1 | Item 2 | Item 3 | Item 4 | Item 5 | Item 6 | Item 7 |
| --- | --- | --- | --- | --- | --- | --- | --- |
| Item 1 | - | **0.7309** | **0.7277** | **0.7335** | 0.6568 | **0.7377** | 0.6202 |
| Item 2 | **0.7305** | - | **0.8748** | **0.7140** | 0.6569 | 0.6984 | 0.6251 |
| Item 3 | **0.7277** | **0.8743** | - | **0.7640** | 0.6603 | **0.7116** | 0.6292 |
| Item 4 | **0.7251** | **0.7096** | **0.7556** | - | **0.7563** | **0.7163** | 0.6561 |
| Item 5 | 0.6448 | 0.6496 | 0.6485 | **0.7559** | - | 0.6862 | 0.6417 |
| Item 6 | **0.7365** | 0.6954 | **0.7107** | **0.7009** | 0.6656 | - | 0.6096 |
| Item 7 | 0.5993 | 0.6107 | 0.6088 | 0.6526 | 0.6403 | 0.5794 | - |

1. Upper diagonal elements are *G*. Lower diagonal elements are *Ф*.

2. The values higher than 0.70 are presented in bold type.

3. The two reliability coefficients of the combination constituting GAD-2 are underlined.


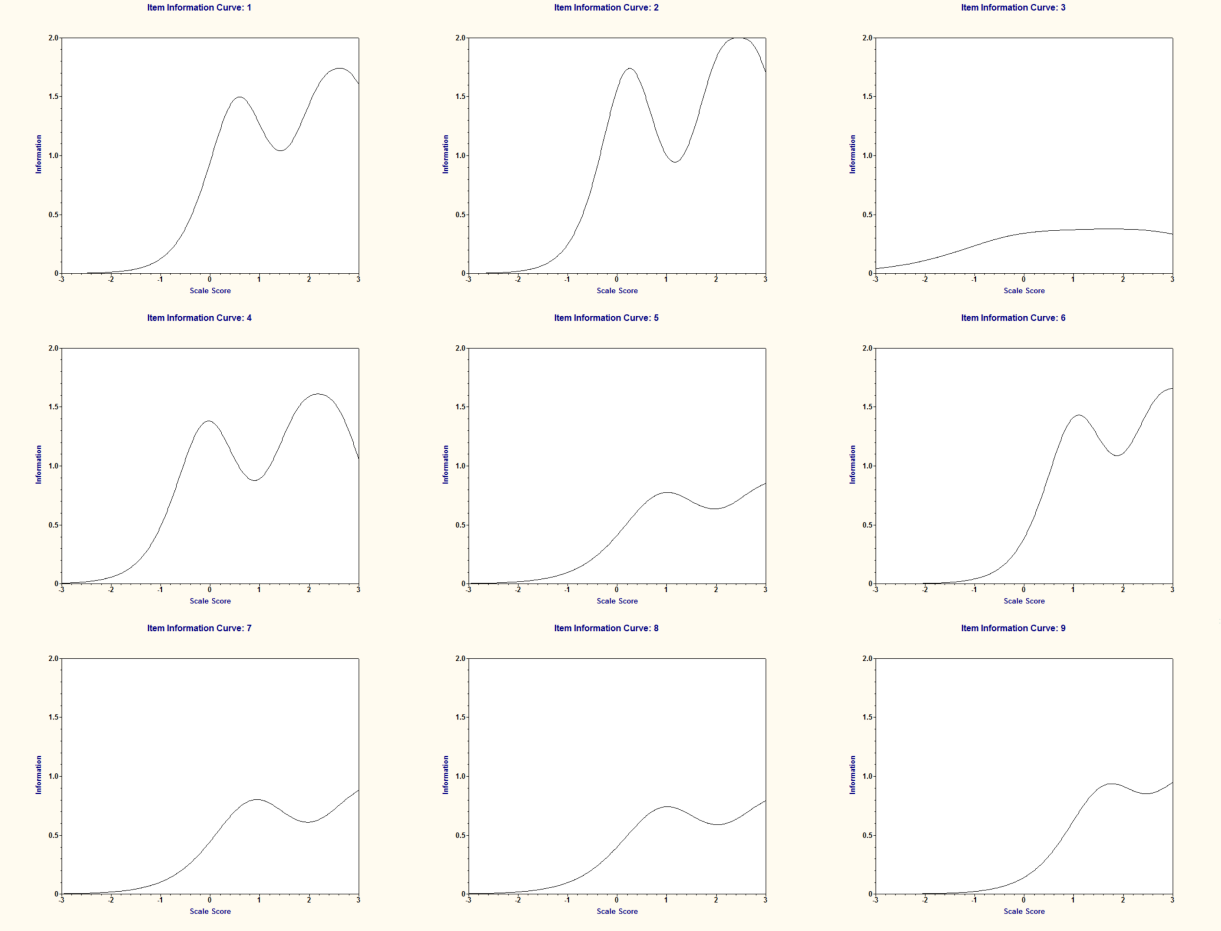


Figure.S2 The item information curve of PHQ-9 for 9 items


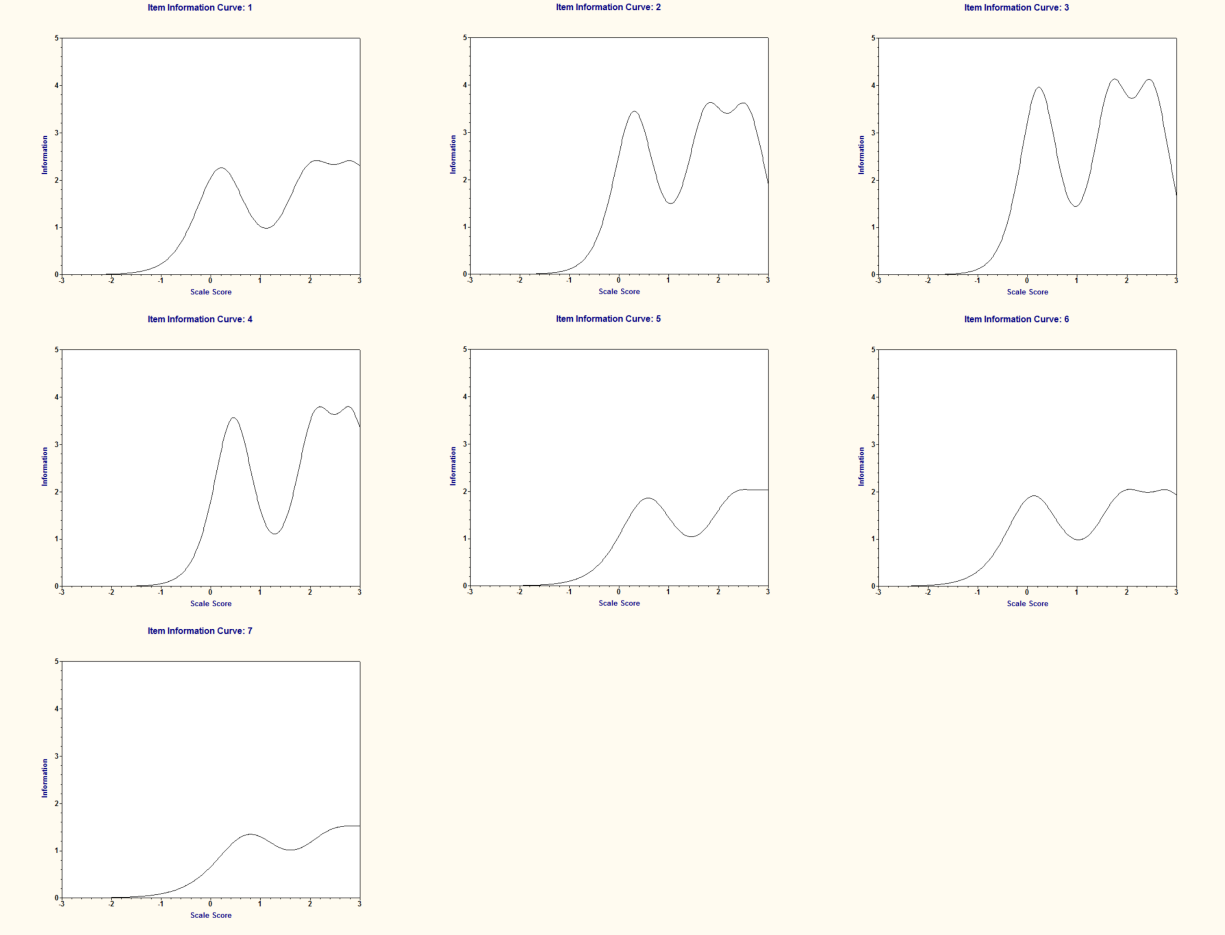


Figure.S3 The item information curve of GAD-7 for 7 items


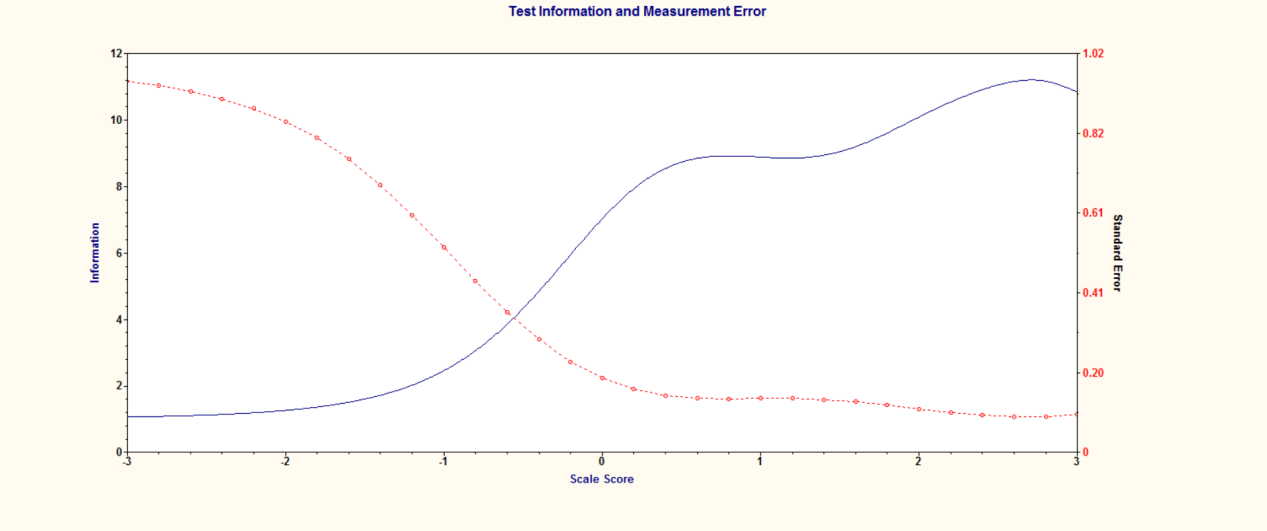


Figure.S4 The test information curve of PHQ-9


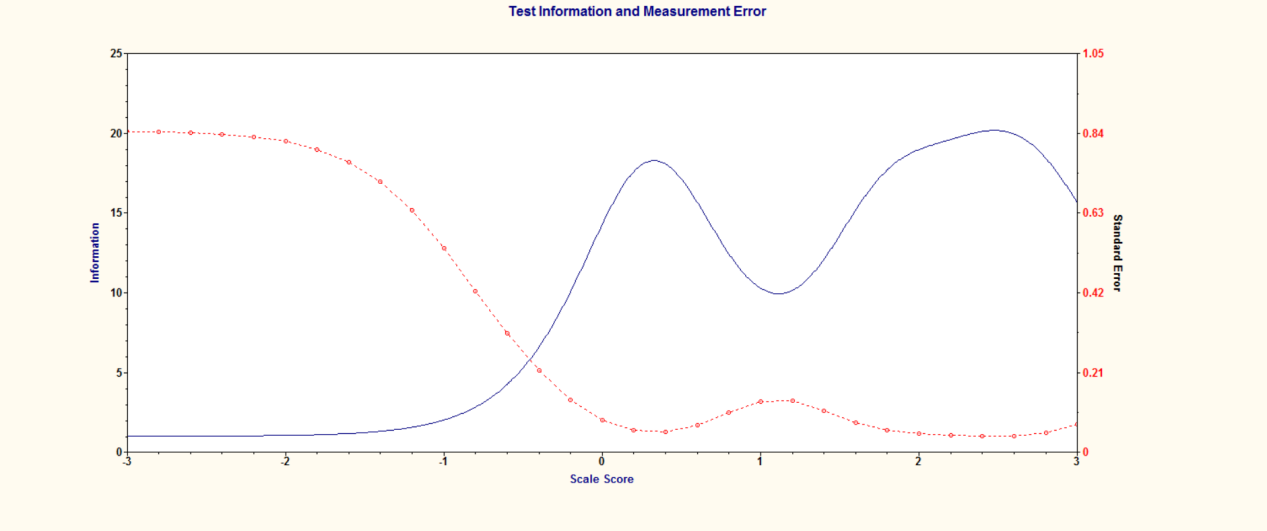


Figure.S5 The test information curve of GAD-7
